# Impact of Nickel Toxicity on Growth, Fruit Quality and Antioxidant Response in Zucchini Squash (*Cucurbita pepo* L.)

**DOI:** 10.3390/plants13172361

**Published:** 2024-08-24

**Authors:** Oumayma Labidi, Rim Kouki, Saida Hidouri, Hana Bouzahouane, Isabel Caçador, Rosa M. Pérez-Clemente, Noomene Sleimi

**Affiliations:** 1 Laboratory RME—Resources, Materials and Ecosystems, Faculty of Sciences of Bizerte, University of Carthage, Jarzouna, Bizerte 7021, Tunisia; oumayma_abidi11@yahoo.fr (O.L.); kouki.rim.kouki@gmail.com (R.K.); 2 LR12SP13, Faculty of Medicine of Monastir, University of Monastir, Av. Avicenne, Monastir 5000, Tunisia; saida.hidouri2020@gmail.com; 3 Faculty of Natural and Life Sciences, University of Mohamed Cherif Messaadia, Souk-Ahras 41000, Algeria; hana_microbiologie@yahoo.fr; 4 Laboratory of Environmental Biosurveillance, Faculty of Sciences, University of Badji Mokhtar, Annaba 23000, Algeria; 5 MARE—Centro de Ciências do Mar e do Ambiente, Faculdade de Ciências, Universidade de Lisboa, Campo Grande, 1749-016 Lisboa, Portugal; micacador@ciencias.ulisboa.pt; 6 Department de Biologia, Bioquímica i Ciències Naturals, Universitat Jaume I, Campus Riu Sec, 12071 Castelló de la Plana, Spain; rosa.perez@uji.es

**Keywords:** *Zucchini*, TME toxicity, non-enzymatic antioxidants, proline

## Abstract

The impact of trace metal elements (TMEs) on plants is one current pollution problem, the severity of which is increasing with industrial development, population growth and inappropriate agricultural practices. The latter can have irreversible effects on ecosystems, including species extinction, trophic chain contamination and altered human health, particularly in the case of consumed plants such as zucchini squash (*Cucurbita pepo* L.). This study aims to investigate the effects of nickel on various physiological and biochemical parameters of zucchini growth, with a particular focus on how this toxic metal impacts the quality of fruit that is consumed by humans. To achieve this, plants aged 45 days were grown for one month on solid media loaded with different concentrations of Ni (0, 100, 300 and 500 µM). The results showed that exposure of plants to Ni resulted in significantly altered growth and higher accumulation of Ni in the shoots (1314 µg·g^−1^ DW) than in roots and fruits. Concerning non-enzymatic antioxidants, the results showed that Ni toxicity significantly increased total polyphenols, especially in shoots at 300 µM Ni, while flavonoid content decreased in the roots and shoots in response to Ni treatment. Our results also show that nickel tolerance in *C. pepo* is ensured by a combination of several mechanisms such as an increase in the content of proline. This species can survive and tolerate, to different degrees, toxic cations at concentrations up to 500 µM but with visible symptoms of toxicity such as chlorosis of the leaves. Indeed, based on thresholds of hyperaccumulation, we can qualify *Cucurbita pepo* as a hyperaccumulator species of nickel.

## 1. Introduction

Soil contamination by trace metal elements (TMEs) is one of the biggest environmental problems worldwide [1]. It presents a risk of toxicity for living beings and humans throughout the food chain [2]. It hampers growth and crop viability depending on the degree of contamination. Studies by Sleimi et al. [3] indicate that many plants from polluted soil were contaminated by TMEs. Likewise, Karimi et al. [4] showed that cultivated crops (*Vicia faba* and *Brassica arvensis*) were contaminated with TMEs such as Pb, Cd and Ni. Altogether, soil contamination finally results in inferior crop quality, harmful effects on human health, contamination of water sources and a negative impact on ecosystem and biodiversity [5].

High levels of geogenic nickel may be transferred from soil to staple crops, resulting in elevated nickel concentrations in cereals. Hseu and Lai [6] reported Ni concentrations ranging from 1.50 to 5.54 mg Kg^−1^ in rice grown in soils with geogenic nickel. Soil pollution is considerable when the compounds introduced alter their function or pose a threat to human health [7]. Health problems may appear following ingestion of food grown on the contaminated site or via inhalation of dust [8].

In plants, some TMEs (Cu, Zn, Ni, Fe, Co) are essential to major physiological processes, in particular, respiration, photosynthesis and macronutrient assimilation. Moreover, some of these TMEs are involved in molecular processes such as the control of gene expression, protein biosynthesis, nucleic acids, phytohormones, chlorophyll and secondary metabolites [9,10].

Environmental contamination by TMEs causes toxicity in biological molecules by producing reactive species, blocking functional groups and replacing basic metal ions [11]. These functional changes translate into visible symptoms at the morphological level [12]. Plant development is reduced, the root system is more compact, and leaves are smaller [13]. The presence of TMEs tends to increase the ratio of root biomass to above-ground biomass, indicating that the plant preferentially maintains its root biomass rather than its above-ground biomass [14]. Root browning, leaf necrosis and chlorosis also occur [15]. Chlorosis is more pronounced in young leaves, while necrosis is more noticeable in older leaves, with higher TME content [16]. However, at low doses, it is possible to observe the opposite effect or hormesis [17].

Nickel is an essential trace element, but at low levels. It is present in high concentrations in ferromagnesian source rocks, in which it partially substitutes for iron or magnesium. It is particularly abundant in the minerals of basic and ultrabasic magmatic rocks and in serpentine ores [18]. Nickel exists in soil mainly in the oxidation state Ni(II). It can occur in the following forms: Ni^2+^, NiOH^+^, Ni(OH)_2_ (aq), Ni(OH)_3_^−^, Ni(OH)_4_^2−^, Ni(OH)_2_(s), NiCO_3_(aq) and NiHCO^3+^ [19]. It can be adsorbed by organic and inorganic matter (Al, Mn, Fe oxides and clay compounds), depending on soil pH [20].

Excessive Ni concentrations in plants can cause iron chlorosis. The nickel content of plants depends on its bioavailability in the soil, the plant species and the specific plant parts analysed. The level of the Ni in plants grown on uncontaminated soil is on the order of 0.05 to 5 mg kg^−1^ dry weight (DW) [21]. Some plants can grow on contaminated or serpentine soils. They can then accumulate more than 1000 mg Kg^−1^ DW, particularly in their leaves [22].

At low concentrations (0.01 to 5 mg Kg^−1^ DW), Ni is considered an essential element. It is involved in the structure of urease, which is responsible for urea hydrolysis in leaves [23]. The toxic effects of nickel and other TMEs mainly manifest through growth inhibition, the extent of which depends on dose, duration of treatment, plant species and chemical form [24,25]. At high concentrations, it becomes toxic: over 10 mg Kg^−1^ for sensitive plants and over 50 mg Kg^−1^ for tolerant plants [26]. This phytotoxicity causes chlorosis [27], reduced growth and metabolic disorders.

Zucchini (*Cucurbita pepo*) is the immature comestible crop of *Cucurbita pepo* spp. It is an important food crop, providing an essential source of minerals, vitamins and fibre [28]. Given the high prevalence of TMEs in agricultural soils, preventives studies and analyzes are therefore necessary as regards the marketing of plants cultivated in contaminated areas. The objective of this research was to investigate the capacity of accumulating Ni in *Cucurbita pepo* and to examine the impact of these elements on the plants’ growth. Our objectives were accomplished by measuring the endogenous amounts of K, Ca and Mg as well as the protein content. Additionally, the contents of proline, total phenols, and flavonoids in the different tissues of the zucchini plant were determined to better understand the adaptation levels and tolerance limits of *Cucurbita pepo* in response to nickel-induced stress.

## 2. Material and Methods

### 2.1. Plant Culture and Treatments

*Cucurbita pepo* (*Cucurbitaceae*) plants used in the experiments were grown from seeds germinated in a perlite and gravel substrate within plastic pots, as described by Labidi et al. [29]. The cultivation took place in a greenhouse under semi-controlled conditions at the Faculty of Science of Bizerte, Tunisia. The seedlings were irrigated with 200 mL of Hewitt’s nutrient solution [30] three times a week. Plants, aged 45 days, were treated for 4 weeks with various Ni concentrations: (a) 0 µM NiCl_2_ (control); (b) 100 µM NiCl_2_; (c) 300 µM NiCl_2;_ and (d) 500 µM NiCl_2,_ added to the nutrient solution.

On the harvest day, plants (aged 75 days) from each treatment group were randomly divided into 2 groups of 5 plants. At the first step, shoots were separated from roots and were washed with cold distilled water and stored in liquid nitrogen for later testing.

To obtain fruits, *Cucurbita pepo* seeds were germinated in peat and then moved to 100% perlite as a substrate. After 2 months, the plants were treated for 10 days with the concentrations of Ni as previously described. This culture was carried out in a greenhouse under semi-controlled conditions at the University of Jaume I, Spain.

The fruits were harvested from 8 weeks after seeding until the 12th week. The harvest was performed by cutting the zucchini fruits to separate them from the plant.

### 2.2. Parameters of Growth

The ratio of shoot/root dry biomass (S/R) and the tolerance index percentage (TI %) [31] were calculated as follows:

S/R = DW shoots/DW roots
TI (%) = DW-treated plants/DW control plants × 100

The relative growth rate (RGR) was determined following Sleimi and Abdelly [32]:

RGR = (ln (W2) − ln (W1)/(t2 − t1)
W is the fresh matter at the beginning of treatment (W1) and at the end (W2).
(t2 − t1) is the duration of this period.

### 2.3. Assay of Nickel and Minerals Elements

The extraction of Ni and mineral element contents from the dry matter of the treated plants was achieved using a mixture of acids: HNO_3_:H_2_SO_4_:HClO_4_ (10:1:0.5; *v/v/v*) at 110 °C for 2 h [29,33]. Afterward, the obtained extracts were diluted with a volume of 50 mL of nitric acid solution (HNO_3_) at 0.5% and filtered. The measurements of Ni, Ca, Mg and K contents was carried out by atomic absorption spectrometry (Perkin Elmer PinAAcle 900T, Waltham, MA, USA). The recorded values were the average of three readings. The limit of detection of Ni, Ca, Mg and K were 0.06, 0.092, 0.0078 and 0.043 mg L^−1^, respectively.

### 2.4. Assay of Total Phenolics and Flavonoids

Phenolic compounds were quantitatively determined using the Folin–Ciocalteu method, which involves the oxidation of a reagent with blue tungsten–molybdenum oxide; the intensity of the blue colour informs the concentration of polyphenols in the extracts [34]. Absorbance was measured at 765 nm, using spectrophotometry (UV/visible, TOMAS UV-1200) and expressed as mg gallic acid equivalents.

Total flavonoid content was determined using the colorimetric method described by Zhishen et al. [35], which was based on the formation of a complex between flavonoids and aluminium trichloride. The flavonoid content was measured at 430 nm, using spectrophotometry (UV/visible, TOMAS UV-1200). Rutine was used as a standard reference for the quantitative estimation of flavonoids.

### 2.5. Assay of Proline

Proline determination followed the method by Bates et al. [36]. Freshly frozen material (50 mg) was ground in 3% sulphosalicylic acid (5 mL) and centrifuged. The supernatant was recovered.

Then, 1 mL of the filtrate was mixed with 1 mL of ninhydrin reagent followed by 1 mL of glacial acetic acid. All samples were incubated at 100 °C for 60 min at 100 °C in a water bath, cooled and centrifuged 5 min at 2000 rpm at 4 °C. Absorbance was read at 520 nm with a spectrophotometer (Thermo Spectronic Genesys 10, Waltham, MA, USA). Total quantification was performed according to a calibration curve prepared with commercial-standard proline.

### 2.6. Assay of Proteins

Frozen zucchini roots and shoots were ground in poly-vinyl-polypyrrolidone (PVP) and homogenized in 50 mM potassium phosphate buffer (pH 7) containing 0.1 mM EDTA (ethylenediaminetetraacetic), 0.1 mM phenyl-methyl-sulphonyl-fluoride (PMSF) acid and 1 mM DTT. The mixture was centrifuged at 12,000× *g* at 4 °C for 30 min. Supernatants were stored at −20 °C for protein and enzyme analysis. The protein content of individual extracts was calculated by the Bradford reaction, utilizing bovine serum albumin (BSA) as reference standard [37].

### 2.7. Statistical Analysis

Samples were tested for a minimum of 3 repetitions, and the mean values and standard error (±) are shown as vertical bars in the graphs. For all data, an analysis of variance (ANOVA) and principal component analysis (PCA) were performed using Statistica 8 and Sigmaplot v. 14.0.0.124 software (Systat Software, San Jose, CA, USA).

## 3. Results

### 3.1. Mineral Nutrition

The addition of Ni^2+^ to irrigation solutions induced a significant increase in Ca^2+^, Mg^2+^ and K^+^ content of roots and shoots (Table 1). Indeed, a large accumulation of Mg^2+^ and K^+^ was noted in the roots with a concentration of 100 µM Ni^2+^ (Table 1).

*Cucurbita pepo* fruits irrigated with the nutrient solution supplemented with different doses of Ni^2+^ showed a decrease in Ca^2+^ concentration, but only with 500 µM Ni. On the other hand, no significant variation in Mg^2+^ and K^+^ nutrient content was observed in the fruit. Nevertheless, at a concentration of 300 µM Ni, there was a decrease in the K^+^ content of the *Cucurbita* fruits (Table 1).

### 3.2. Nickel Content

**Figure 1:**
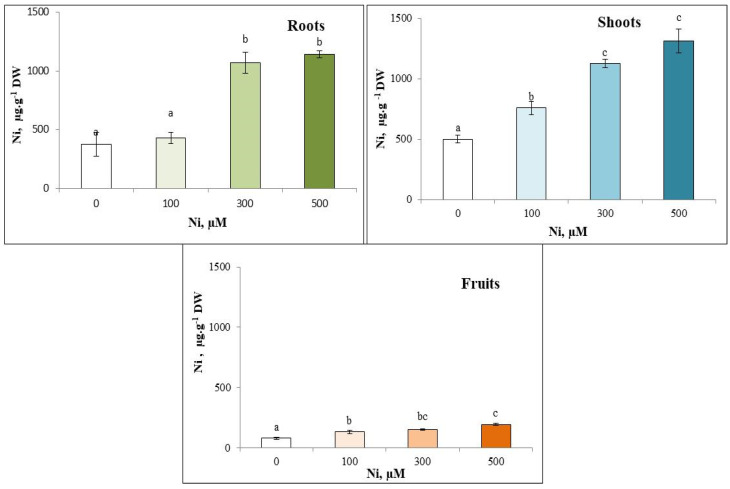
Variation in nickel content in the roots, shoots and fruits of zucchini with different concentrations of Ni (0, 100, 300 and 500 µM). Mean values of 6 replicates. Values with different letters are significantly different (*p* ≤ 0.05) according to Tukey’s HSD test.

The results showed that Ni^2+^ content increased in different parts of the zucchini plants with increasing Ni^2+^ concentrations in the culture medium. The endogenous concentration of Ni^2+^ ions increased more significantly in the roots and shoots compared to the fruits. Maximum accumulation was observed at 500 µM, reaching 1140.07 µg·g⁻^1^ DW in the roots, 1314 µg·g⁻^1^ DW in the shoots, and 195 µg·g⁻^1^ DW in the fruits (Figure 1).

### 3.3. Plant Morphology and Growth

**Figure 2:**
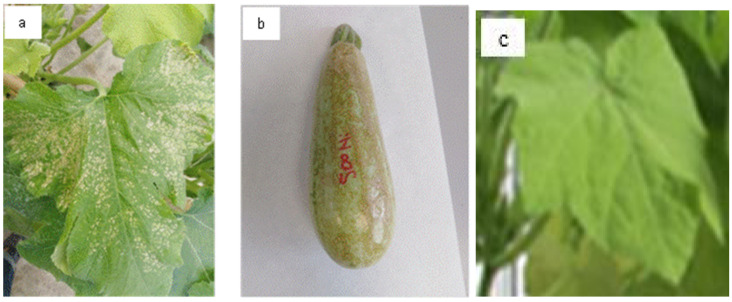
Leaf chlorosis (**a**), fruit damage (**b**) in *Cucurbita pepo* exposed to high concentrations of Ni^2+^ (500 µM) after 21 and 60 days of treatment, respectively. (**c**): a leaf of the control plant.

From the 3rd week of treatment, Ni affected plant development and growth. Indeed, chlorosis appeared on *Cucurbita pepo* leaves treated with Ni^2+^ compared to the leaves of control plants (Figure 2a,c). At the end of the treatment, these toxicity symptoms were more pronounced and accompanied by leaf abscission.

Concerning fruits (60 days of treatment), brown spots were observed on *Cucurbita pepo* fruit treated with Ni^2+^ at 500 µM (Figure 2b).

The presence of Ni^2+^ in the culture medium reduced dry biomass production in *Cucurbita pepo*, with the dry weight decreasing from 186.9 mg to 103.4 mg and from 2699.9 mg to 442.5 mg, respectively, in roots and shoots with 500 µM Ni (Table 2).

The calculation of the S/R ratio shows a significant decrease with Ni^2+^ treatment, indicating that Ni^2+^ affects the shoots more than the roots. This suggests that Ni^2+^ disrupts growth more in the shoots relative to the roots, potentially leading to imbalances in plant development (Table 2).

The estimation of the tolerance index for the whole plant showed a decline as Ni concentrations in the culture medium increased. This decrease indicates that higher levels of Ni reduce the plant’s ability to tolerate stress, impacting its overall health and growth (Table 2).

**Figure 3:**
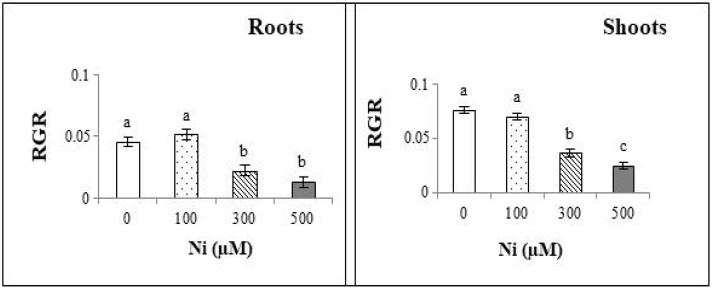
Relative growth rate in the roots and the shoots of *Cucurbita pepo* exposed to different concentrations of Ni^2+^ (0, 100, 300 and 500 µM). Mean values of 10 replicates. Values with different letters are significantly different (*p* ≤ 0.05) according to Tukey’s HSD test.

The relative growth rate (RGR) of both shoots and roots shows a notable decrease with increasing Ni^2+^ concentrations in the irrigation solutions (Figure 3). This reduction indicates that higher Ni^2+^ levels significantly hinder the growth of both plant parts.

### 3.4. Water Content

**Figure 4:**
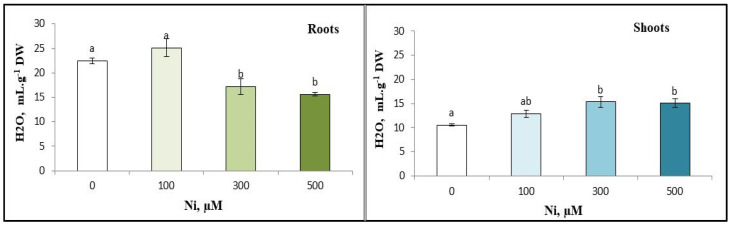
Variation in water content of the roots and the shoots of zucchini with different concentrations of Ni (0, 100, 300 and 500 µM). Mean values of 10 replicates. Values with different letters are significantly different (*p* ≤ 0.05) according to Tukey’s HSD test.

The results showed a significant decrease in root water content with increasing Ni^2+^ concentrations, indicating impaired water retention. In contrast, shoot water content increased significantly with higher Ni^2+^ levels, suggesting improved tissue hydration in the shoots. This suggests a shift in water balance within the plant due to Ni^2+^ treatment. (Figure 4).

### 3.5. Total Polyphenol (TP) Content

**Figure 5:**
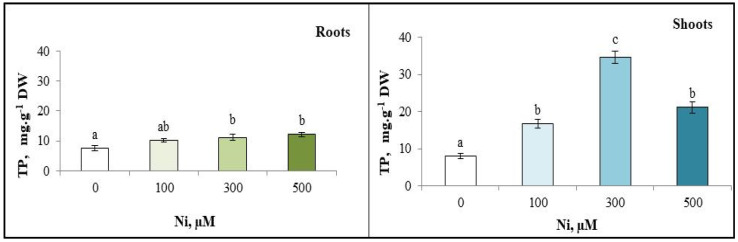
Variation in total polyphenols (TP) content of the roots and the shoots of zucchini with different concentrations of Ni (0, 100, 300 and 500 µM). Mean values of 6 replicates. Values with different letters are significantly different (*p* ≤ 0.05) according to Tukey’s HSD test.

The results of the total polyphenol assessment revealed that Ni induced a significant increase in total polyphenol content of all organs of *C. pepo*. In fact, the polyphenol content increased in the roots and shoots according to the Ni^2+^ concentration in the medium. The highest levels of TP in *C. pepo* were found in the shoots of plants exposed to 300 µM Ni^2+^ (34.6 mg·g^−1^ DW) compared with the control (7.98 mg·g^−1^ DW) (Figure 5).

### 3.6. Flavonoid Content

**Figure 6:**
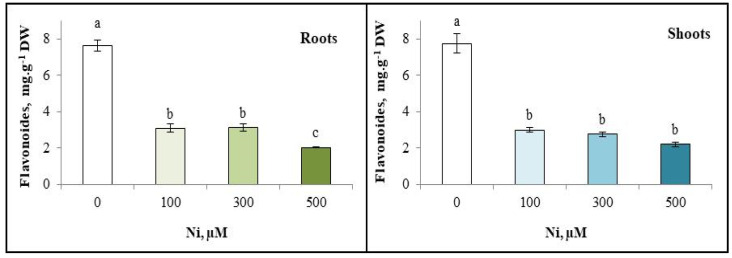
Variation in flavonoid content of the roots and the shoots of zucchini with different concentrations of Ni (0, 100, 300 and 500 µM). Mean values of 6 replicates. Values with different letters are significantly different (*p* ≤ 0.05) according to Tukey’s HSD test.

Analysis of the variation in flavonoid content of the roots and shoots of *Cucurbita pepo* shows the same behaviour. In fact, these contents decreased as a function of the Ni concentration in the irrigation solutions. However, the higher decrease in flavonoid content was observed in the presence of 500 µM Ni^2+^, with a 73% decrease in *C. pepo* roots (Figure 6).

### 3.7. Proline Content

**Figure 7:**
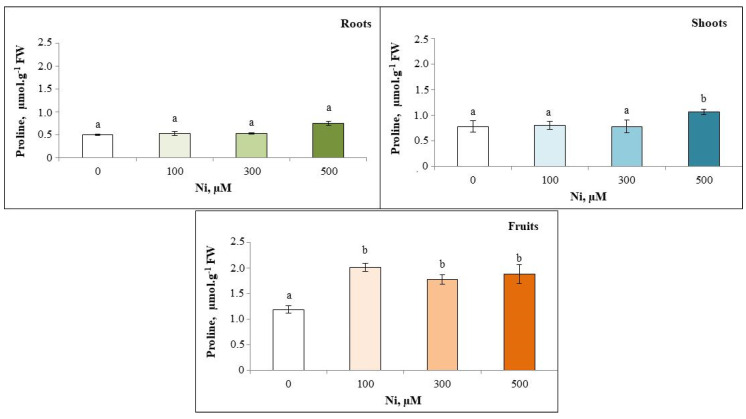
Variation in proline content of the roots, shoots and fruits of zucchini with different concentrations of Ni (0, 100, 300 and 500 µM). Mean values of 6 replicates. Values with different letters are significantly different (*p* ≤ 0.05) according to Tukey’s HSD test.

The study of proline content during the vegetative stage in the shoots shows that the addition of Ni^2+^ to the irrigation solutions induces a significant accumulation of proline in *C. pepo* at high doses (500 µM Ni^2+^). However, Ni-induced stress did not show any significant variations compared with the control in the roots (Figure 7).

### 3.8. Total Protein Content

**Figure 8:**
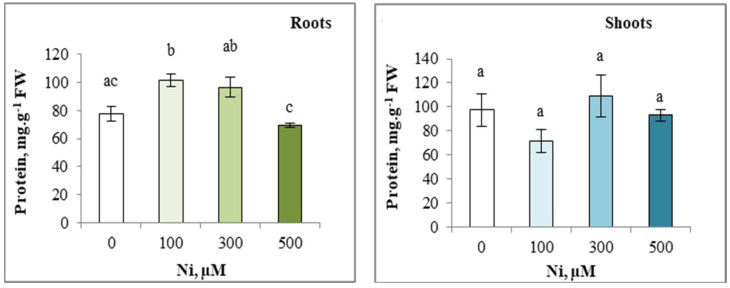
Variation in total protein content of the roots and the shoots of zucchini with different concentrations of Ni (0, 100, 300 and 500 µM). Mean values of 6 replicates. Values with different letters are significantly different (*p* ≤ 0.05) according to Tukey’s HSD test.

The results in Figure 8 show that the addition of the different doses of Ni^2+^ to the irrigation solution did not significantly affect the total protein content of the shoots. However, an accumulation of total protein in the roots was observed with the 100 and 300 µM Ni^2+^ solution (Figure 8).

### 3.9. Principal Component Analysis (PCA) 

PCA was carried out in order to study the impact of Ni on the different studied parameters in *C. pepo*, as well as the correlation between these parameters.

**Figure 9:**
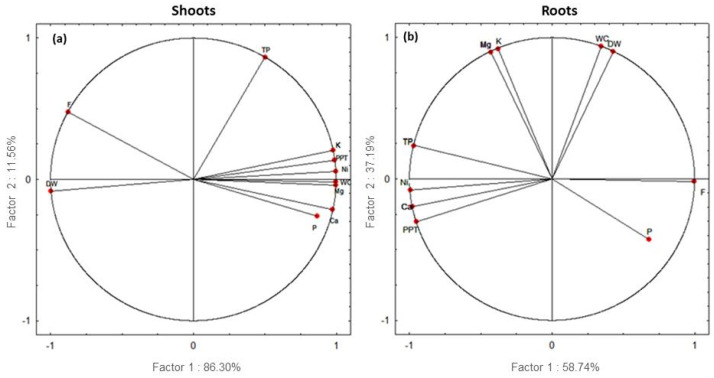
Correlation circle from the PCA of nickel (Ni) content, dry weight (DW), water content (WC), potassium (K), calcium (Ca), magnesium (Mg), proline (P), total protein (TP) total polyphenols (PPT) and flavonoids (F). Data of the shoots (**a**) and the roots (**b**) of *Cucurbita pepo* plants subjected to increasing doses of Ni (0, 100, 300, 500 µM).

Our results showed a high significance; in the shoots of the plants treated with Ni, total variance reached 97.86%, while in the roots, total variance was 95.93%. In the shoots (a), Ni content was negatively correlated with water content, mineral nutrient content, total polyphenols and proline (Figure 9). The data revealed that, in the roots (b), Ca content, total protein and total polyphenols were positively correlated with Ni content (Figure 9). However, DW and Ni content presented negative correlation with flavonoids.

## 4. Discussion

Cultured seedlings exhibit different growth behaviours in response to the presence of TMEs in the culture medium. Our results showed significantly reduced biomass production in *Cucurbita pepo* irrigated with Ni^2+^ supplemented nutrient solution. Indeed, plant growth at the aerial part severely decreased by 84% in *Cucurbita pepo* grown in the presence of 500 µM Ni^2+^. These seedlings also showed a significant 44% reduction in root growth in the presence of 500 µM Ni. The same results were observed in *Oryza sativa* in the presence of 200 and 400 µM Ni [38]. Similarly, Gajewska and Sklodowska [39] showed that wheat shoot biomass was reduced by 20 and 26% with Ni concentrations of 100 µM and 200 µM, respectively. According to Palacios et al. [40], tomato plant vegetative growth and fruit production decreased significantly with increasing levels of Ni in the nutrient solution. Indeed, plants’ Ni uptake reduces shoot and root growth and leaf area [9].

Tolerance index (TI) defines the ability of plants to grow well and tolerate high metal concentrations. Our results show that TI values decreased in *C. pepo* in the presence of Ni in the culture medium. Bouslimi et al. [41] reported a decrease in TI in *Cakile maritima* under the effect of barium.

The depressive effects of Ni on plant growth are due to several factors. Nickel disrupts enzymatic activities [42] and hormonal balances, affecting cell division and increasing stress hormones like abscisic acid [43]. Nickel also displaces essential nutrients like iron and magnesium, causing deficiencies that hinder photosynthesis. Additionally, Ni generates oxidative stress, damaging cellular components. These combined effects significantly disrupt plant growth and development [44].

Disruption of water parameters has also been reported in plants exposed to metal stress [45] (Poschenrieder et al., 1989). Water disruption in plants under metal stress has been documented [45] (Poschenrieder et al., 1989). Our findings show an increase in water content of aerial parts but a decrease in the roots of *C. pepo* under Ni^2+^ stress. Kouki et al. [46] reported similar reductions in water content under metal stress. Barcelo and Poschenrieder [47] found that metal stress blocks water transport. Ni toxicity reduces water content and transpiration rates, even at low doses [48]. These disturbances cause stomatal closure [49] and inhibit absorptive hair formation, reducing water and nutrient uptake.

In the presence of Ni, the content of mineral nutrients in plant organs may increase, decrease or stay even, and thus may disrupt the uptake and distribution of essential nutrients to different parts of the plant [4,44].

In the present study, the addition of Ni^2+^ to the culture medium induced a significant increase in K^+^ levels in the aerial and root parts of *C. pepo*. The improvement in potassium nutrition has been mentioned in several research studies.

Indeed, increased root K^+^ levels were observed in tomatoes [40] and maize plants exposed to Ni [50], though some studies reported decreased K^+^ under metal stress, possibly due to differences in species and methods [51].

Magnesium, the central atom in chlorophyll, activates many enzymes [52]. Magnesium levels decrease under metal stress as TMEs replace Mg^2+^ in chlorophyll, causing its destruction [53] (Rizwan et al., 2019). Reduced Mg^2+^ has been noted in tomatoes exposed to Ni [40]. TMEs alter nutrient uptake by changing membrane permeability and nutrient transport [54,55,56]. Conversely, some studies found increased Mg^2+^ under metal stress. Magnesium deficiency could mitigate TME toxicity by maintaining ferric status and increasing iron in Ni^2+^-treated leaves, preventing chlorosis and restoring chlorophyll [57]. Mg^2+^ may also enhance antioxidant capacity for detoxification and photosynthetic protection.

One of the probable mechanisms for decreasing the uptake of macro- and micronutrients relies on competition for the common binding sites due to the comparable ionic radii of Ni^2+^ and other cations. The decline in nutrient uptake may also result from Ni-induced metabolic disorders that affect the structure and enzyme activities of cell membranes [44].

Calcium nutrition in plants is generally affected by metal stress. Our results showed that the addition of Ni^2+^ to the culture medium increased Ca^2+^ levels in the roots and aerial parts of *C. pepo*. This aligns well with the work of Sghaier et al. [58], who showed that Ca^2+^ uptake was increased in *Tamarix gallica* under the effect of aluminium. Calcium has been reported to attenuate the adverse effects of TMEs on plants [59]. The decrease in Ni^2+^ toxicity by Ca^2+^ may be due to the fact that plasma membrane surfaces are generally negatively charged, and high Ca^2+^ concentrations tend to neutralize them and thus minimize the toxic effect of Ni^2+^.

Nickel is known to be readily absorbed by most species, as a divalent cation. It can compete with other cations, including Ca^2+^, Mg^2+^, Fe^2+^ and Zn^2+^ [60]. Nickel levels in the roots and aerial parts of *C. pepo* increased with increasing Ni concentration in the culture medium. Consequently, Ni phytoavailability depends on both Ni level in solution and treatment time [40]. Moreover, Sleimi et al. [61], show that the increase in TME content in roots and aerial parts is proportional to the increase in TME concentration in the irrigation solution in *Cucumis sativus*. Our results show that *C. pepo* can accumulate 1314 µg·g^−1^ DW Ni^2+^ in its aerial parts. These data suggest that *C. pepo* has a strong capacity to accumulate and tolerate these TMEs at the tissue level.

According to Reeves and Baker [62], a species is considered a hyperaccumulator if it accumulates more than 1000 µg·g^−1^ DW Ni^2+^ in its aerial parts. However, Ni hyperaccumulators can store more than 1000 μg·g^−1^ Ni DW in their tissues when grown on a nickel-rich substrate [10]. Furthermore, we can qualify *C. pepo* as a Ni^2+^ hyperaccumulative species.

In response to abiotic stresses, the biosynthesis of secondary metabolites generally increases in plants. Phenolic compounds confer, in fact, a higher tolerance to plants against various abiotic stresses, such as TME, salinity, drought, temperature, pesticides and UV radiation [63]. Indeed, polyphenol accumulation could play a key role in plant balance and adaptation [64]. This accumulation is more important in the leaves than in the roots of the plant; in this respect, several authors, such as Jonnala et al. [65] and Chaib et al. [66], have shown the richness of cereal leaves with polyphenols, which possess great antioxidant power.

Our results show that the production of total polyphenols increases under Ni^2+^-induced metal stress in the aerial parts and roots of *C. pepo*. Indeed, a significant threefold increase was observed in plants grown in the presence of 500 µM Ni^2+^ compared with control plants. These results are consistent with Rastgoo et al. [67], who observed an increase in polyphenols under metal stress (Pb, Co, Cd, Ag) in *Aeluropus littoralis*; the same behaviour was reported in lead-stressed *Raphanus sativus* [68].

Stimulating the biosynthesis of phenolic compounds helps protect plants from oxidative stress due to metal exposure [69]. These compounds, with antioxidant properties, scavenge free radicals, reducing cell membrane peroxidation and protecting plant cells. Phenols also enhance membrane stability, contributing to cell rigidity and creating barriers against heavy metals [70]. They chelate metal ions and inhibit the Fenton reaction, promoting plant growth under metal stress [71].

Ma et al. [72] found a direct correlation between TMEs in the culture medium and the expression of phenylalanine ammonia-lyase (PAL), a key enzyme in phenolic compound biosynthesis in response to stress [73]. This explains the accumulation of phenolic acids in TME-stressed plants. Handa et al. [69] showed that increased PAL expression leads to higher levels of total phenols and flavonoids in *Brassica juncea* under Cr stress. Similarly, Pb stimulates PAL activity in soybean [74]. The accumulation of phenolic compounds results from the regulation of phenylpropanoid enzyme biosynthesis, including PAL, chalcone synthase, shikimate dehydrogenase, cinnamyl alcohol dehydrogenase and polyphenol oxidase [75], driven by the modulation of gene transcript levels under metal stress [69].

On the other hand, flavonoid accumulation has been demonstrated in many species and under different abiotic constraints [76,77]. Flavonoid production differs from that of TPP in the aerial parts and roots of *C. pepo* grown in the presence of Ni. Indeed, the flavonoid content of *C. pepo* leaves and roots decreases proportionally with nickel concentration in irrigation solutions; this could be explained by plant resistance to these concentrations [78]. Plant response to metal stress thus varies from one concentration to another [79]. In general, the presence of Ni in irrigation solutions inhibits flavonoid synthesis. Murch et al. [80] reported reduced flavonoid levels in plants exposed to Ni and Cu. Similarly, reduced flavonoid levels were observed in *Atriplex canescens* roots exposed to Cd [81]. However, Bouslimi et al. [77] report an accumulation of flavonoids in different parts of the plant under stress induced by lanthanum. Similarly, Bretzel et al. [82] have shown that flavonoid content increases in *Taraxacum officinale* grown in the presence of Cr, Pb, Cu, Ni or Zn.

Flavonoids are good inhibitors of enzymes responsible for free radical production, such as xanthine oxidase, cyclooxygenase and lipooxygenase [76].

Actually, flavonoids enhance TME chelation, reducing harmful radicals in plant cells [83]. They scavenge H_2_O_2_ and are crucial in the phenolic/ascorbate peroxidase cycle [84]. Their antioxidant properties come from hydroxyl groups that stabilize radicals [85]. Berni et al. [86] noted that flavonoids act secondarily in plant defence after phenolic acids, conserving energy.

Proline levels in *Cucurbita pepo* increase with Ni concentration more in fruits and leaves than roots, suggesting synthesis in leaves and migration to roots [87]. This is consistent with high proline in *Vaccinium myrtillus* leaves under Zn stress [88], and in *Pinus sylvestris* needles with TMEs [89]. Hadjadj et al. [90] link increased proline to abiotic stress tolerance.

Proline accumulation results from inhibition of its oxidation, increased protein catabolism and/or synthesis of this amino acid. This accumulation protects the cell membrane and is involved in osmotic adjustment, metal chelation and detoxification, enzyme protection, regulation of cytosolic acidity, stabilization of the protein synthesis machinery and scavenging of reactive oxygen species [91].

Overall, the species most sensitive to stress react by accumulating proline more rapidly. On the other hand, those that have proved tolerant show relative stability or low accumulation of their proline content [92]. Brinis and Belkhoudja [93] justified the reduced levels of proline in stressed plants by the fact that the level of stress does not seem to have triggered the proteolysis required to obtain significant quantities of proline, and consequently there is no response to the point of envisaging a possible blockage of the plant’s metabolic activity. Thus, Spormann et al. [94] clarify the implications of proline accumulation in plants under abiotic stress induced by soil degradation factors, reinforcing the idea that proline quantification should not be employed as a sole indicator of stress sensitivity or resilience but rather complemented with further biochemical and physiological endpoints. The decrease in proline in plants subjected to TME-induced stress has been examined in several species, *Raphanus sativus* stressed by zinc [95], *Brassica juncea* subjected to high doses of lead and cadmium [96], and *Abelmoschus esculentus* root’s subjected to 100 and 200 µM doses of Ba [92].

The results of the present study showed an increase in protein levels in *C. pepo* with increasing the as applied doses of Ni. Dridi et al. [97] reported that Pb application resulted in an increase in nitrogen content of shoots and roots of *Helianthus annuus*. This protein accumulation could be the consequence of the plant’s synthesis of defence proteins against this metal stress [98], including proteins involved in maintaining the cell’s redox status, such as ascorbate, or in metal sequestration (GSH, PC) [99] (Yadav, 2010). Other studies report the induction of heat shock-specific proteins under metal stress, demonstrating their role in the adaptive response [100].

## 5. Conclusions

In conclusion, this study demonstrates that Ni concentrations induce notable changes in physiological and biochemical parameters in the roots, shoots, and fruits of zucchini plants (*Cucurbita pepo*). While the doses of Ni used in this study are not highly toxic to zucchini, the plant exhibits significant adaptability, evidenced by its survival and ability to accumulate substantial amounts of Ni in the leaves. This accumulation classifies *C. pepo* as a hyper-accumulator species.

Our findings highlight that increases in proline content play a crucial role in the plant’s response to Ni toxicity. Although *Cucurbita pepo* can tolerate Ni levels up to 500 µM, with observable symptoms such as leaf chlorosis, the accumulation of Ni in the fruits poses a risk of contaminating the food chain. Consequently, it is advisable to utilize *C. pepo* primarily for phytoremediation purposes, specifically to clean soils contaminated with nickel, rather than for consumption. This approach can mitigate the potential entry of Ni into the trophic chain and address the broader issue of TMEs in agricultural ecosystems.

## Figures and Tables

**Table 1:** Variation in calcium (Ca), magnesium (Mg) and potassium (K) contents in the roots, shoots and fruits of zucchini with different concentrations of Ni (0, 100, 300 and 500 µM). Mean values of 6 replicates. Values with different letters are significantly different (*p* ≤ 0.05) according to Tukey’s HSD test.

Treatments (Ni, µM)	Ca^2+^, mg·g^−1^ DW			Mg^2+^, mg·g^−1^ DW			K^+^, mg·g^−1^ DW		
	Roots	Shoots	Fruits	Roots	Shoots	Fruits	Roots	Shoots	Fruits
0 µM	54.51 ± 1.38 ^a^	55.90 ± 2.19 ^a^	13.58 ± 1.01 ^a^	7.22 ± 0.20 ^a^	11.48 ± 0.40 ^a^	12.66 ± 0.70 ^a^	11.33 ± 0.52 ^a^	17.63 ± 0.66 ^a^	71.92 ± 3.16 ^a^
100 µM	67.67 ± 4.48 ^b^	75.01 ± 2.21 ^b^	11.94 ± 1.11 ^ab^	23.55 ± 0.98 ^b^	17.31 ± 0.51 ^b^	14.01 ± 0.76 ^a^	73.44 ± 2.26 ^b^	26.74 ± 1.00 ^b^	73.86 ± 3.69 ^a^
300 µM	71.15 ± 1.43 ^b^	84.08 ± 2.59 ^b^	15.20 ± 1.57 ^a^	9.16 ± 0.30 ^a^	11.92 ± 1.11 ^c^	12.48 ± 2.02 ^a^	15.47 ± 1.26 ^a^	53.40 ± 2.78 ^c^	58.13 ± 3.53 ^a^
500 µM	71.25 ± 3.13 ^b^	81.26 ± 3.28 ^b^	7.64 ± 0.89 ^b^	7.18 ± 0.38 ^a^	9.15 ± 0.29 ^a^	12.04 ± 1.60 ^a^	10.70 ± 0.64 ^a^	19.35 ± 0.50 ^a^	73.77 ± 6.72 ^a^

**Table 2:** Variation in dry weight (DW), shoot/root ratio (S/R) and tolerance index (TI) of zucchini with different concentrations of Ni (0, 100, 300 and 500 µM). Mean values of 10 replicates. Values with different letters are significantly different (*p* ≤ 0.05) according to Tukey’s HSD test.

Treatments (Ni, µM)	DW (g)		S/R	TI (%)
	Roots	Shoots		
0	186.9 ± 9.00 ^a^	2699.9 ± 173.8 ^a^	15.1 ± 1.8 ^a^	
100	204.9 ± 15.2 ^a^	1901.9 ± 118.0 ^b^	9.8 ± 1.0 ^b^	73.9 ± 6
300	127.9 ± 10.5 ^b^	617.9 ± 49.9 ^c^	5.1 ± 0.5 ^c^	22.2 ± 1.6
500	103.4 ± 7.2 ^b^	442.5 ± 33.5 ^c^	4.4 ± 0.4 ^c^	14.6 ± 1.3

## Data Availability

Data are contained within the article.
